# Stallion spermatozoa express LDH isoforms A, B, and C, with LDHC playing a crucial role in sustaining sperm viability

**DOI:** 10.1530/REP-24-0436

**Published:** 2025-06-05

**Authors:** Laura Becerro-Rey, Francisco E Martín-Cano, Antonio Silva-Rodríguez, Cristina Ortega-Ferrusola, Eva da Silva-Álvarez, Cándido Ortiz-Placín, Jose Antonio Tapia, María Cruz Gil, Fernando J Peña

**Affiliations:** ^1^ Laboratory of Equine Reproduction and Equine Spermatology, Veterinary Teaching Hospital, University of Extremadura, Cáceres, Spain; ^2^ Facility of Innovation and Analysis in Animal Source Foodstuffs, University of Extremadura, Cáceres, Spain; ^3^ Department of Physiology, University of Extremadura, Cáceres, Spain

**Keywords:** stallion, spermatozoa, LDH, lactate, pyruvate, NAD^+^

## Abstract

**In brief:**

Three isoforms of lactate dehydrogenase (LDH) – LDHA (cytoplasmic), LDHB (mitochondrial), and LDHC (flagellar) – have been identified and localized in stallion spermatozoa. Functional inhibition assays indicate that these three isoforms constitute a lactate shuttle of crucial importance for sperm function.

**Abstract:**

Stallion spermatozoa use different energy sources; while oxidative phosphorylation predominates, glycolysis and beta-oxidation of fatty acids are also present. Glycolysis depends on the availability of NAD^+^ as an electron acceptor. During glycolysis, NAD^+^ is reduced to NADH. To ensure glycolysis can continue, NAD^+^ must be regenerated. This regeneration typically occurs when NADH donates its electrons to the electron transport chain (specifically at Complex I), where it is oxidized back to NAD^+^. If mitochondria are damaged, the regeneration of NAD^+^ may be compromised, leading to reduced glycolysis and altering sperm metabolism. However, alternative ways to regenerate NAD^+^ may be present. We hypothesized that aerobic glycolysis is present in the stallion spermatozoa as a backup mechanism to regenerate NAD^+^. We incubated spermatozoa in two Tyrode’s modified media with either 67 mM glucose and 1 mM pyruvate or 67 mM glucose and 10 mM pyruvate. The addition of 10 mM pyruvate improved sperm motility (*P* < 0.001). Spermatozoa incubated in 67 mM glucose and 1 mM pyruvate for 3 h at 37°C showed a significant decrease in motility (58.1 ± 1.8% vs 81.2 ± 1.8%, *P* < 0.0001). In contrast, spermatozoa incubated in 67 mM glucose and 10 mM pyruvate retained motility (77.1 ± 1.4%), viability, and mitochondrial membrane potential. We studied the metabolic proteome and metabolome and identified three different isoforms of the enzyme lactate dehydrogenase (LDH), LDHA (cytosolic), LDHB (mitochondrial, with higher affinity for pyruvate), and LDHC (cytosol, motile cilium). Functional experiments using a specific inhibitor of LDHC demonstrated that this isoform may be essential for sperm function. We concluded that activation of aerobic glycolysis in a high-glucose medium improves sperm survival through the regeneration of NAD^+^.

## Introduction

The equine industry relies heavily on the trade of semen doses. Sperm conservation, utilizing refrigerated and frozen-thawed spermatozoa, is essential for artificial insemination and other reproductive technologies, including *in vivo* and *in vitro* embryo production. Semen extenders are vital for preserving sperm viability and fertility over extended periods (Clulow & Gibb 2022). Although supraphysiological glucose concentrations can be detrimental to the equine spermatozoa (Ortiz-Rodriguez *et al.* 2021), most commercial extenders for equine semen are formulated with high glucose concentrations as the main energy source (Kenney *et al.* 1976, Batellier *et al.* 1998). Our understanding of stallion spermatozoa has evolved in key areas, with important implications for sperm biotechnologies, particularly redox regulation and metabolism (Gibb *et al.* 2014, Peña & Gibb 2022). Recent research has highlighted the high dependence of stallion spermatozoa on ATP production through oxidative phosphorylation in the mitochondria (Gibb *et al.* 2014, Davila *et al.* 2016) and identified pyruvate and lactate as preferred metabolic substrates (Gibb *et al.* 2015, Darr *et al.* 2016, Peña *et al.* 2022, Ramirez-Agamez *et al.* 2024, Peña *et al.* 2025). These findings are prompting a reassessment of long-standing assumptions regarding stallion sperm biology and spurring the development of more physiological extenders for improved stallion sperm preservation (Clulow & Gibb 2022). Proteomics and metabolomics are emerging as potent tools to further elucidate the biology of stallion spermatozoa (Peña *et al.* 2024*b*). Under certain conditions, supraphysiological glucose concentrations may impact the sperm proteome; this effect has been well-documented in studies on diabetic conditions in humans (Brownlee 2001, Barati *et al.* 2016, Aluksanasuwan *et al.* 2020, Sagmeister *et al.* 2021, Li *et al.* 2024). Recent research has also heightened interest in pyruvate (reviewed in Peña *et al.* (2025)). Beyond serving as an energy substrate, pyruvate may have additional benefits, such as regenerating NAD^+^ through its reduction to lactate, which is crucial for glycolysis, various stages of the tricarboxylic acid (TCA) cycle, and pyruvate oxidation to acetyl-CoA. The reactions involving the reduction of pyruvate to lactate and the oxidation of lactate to pyruvate are catalyzed by diverse isoforms of the enzyme lactate dehydrogenase (LDH). Pyruvate may also provide mitochondrial protection after conversion to lactate (Cai *et al.* 2023). Based on this evidence, we hypothesize that high glucose concentrations in the medium could induce functional changes in stallion spermatozoa, particularly affecting the metabolic proteome, and that pyruvate could help prevent these alterations through mechanisms involving the action of the LDH and potentially the existence of a lactate shuttle, transporting lactate into the mitochondria (de Burgos *et al.* 1978, Burgos *et al.* 1982, Gallina *et al.* 1994). We used proteomics, metabolomics, and multiparametric flow cytometry to evaluate sperm functionality under these conditions and the potential existence of a functional lactate shuttle in the stallion spermatozoa.

## Materials and methods

### Reagents and media

Chemicals were purchased from Sigma Aldrich (Madrid, Spain), (ethylamino) (oxo)acetic acid was purchased from Cymit Química, Barcelona, Spain. Anti-LDHA, LDHB and LDHC antibodies were purchased from Genetex (USA). All other reagents for flow cytometry were purchased from Thermo Fisher (USA). ViaKrome 808 Fixable Viability Dye was purchased from Beckman Coulter (USA). Ultra-pure deionized water (>18.2 MΩ·cm) was produced from a Millipore Milli-Q Gradient system (Millipore, USA).

### Semen collection and processing

Semen was collected from four fertile stallions maintained according to the institutional and European animal care regulations (Law 6/2913 June 11 and European Directive 2010/63/EU). Routine semen collection from stallions used for commercial semen production did not require ethical approval. Ejaculates were collected using a pre-warmed, lubricated Missouri model artificial vagina following standard veterinary practices. After collection, the ejaculate was immediately extended 1:1 in Tyrode’s media, evaluated for sperm motility and concentration, and processed in the adjacent laboratory. Colloidal centrifugation (Morrell *et al.* 2011) was performed to remove dead spermatozoa and potential contaminating cells from the ejaculate. The pellet was re-extended to a final concentration of 25 × 10^6^ spermatozoa/mL in different variants of Tyrode’s media modified using different concentrations of glucose and/or pyruvate, glucose 67 mM/pyruvate 1 mM (G67-1P) and glucose 67 mM/pyruvate 10 mM (G67-10P). The pH was adjusted to 7.4 and osmolarity to 310 mOsm/kg. The media recipes are provided in Supplementary Table 1 (see section on Supplementary materials given at the end of the article).

### Experimental design

Ejaculates from four different stallions were split, with three replicates each (*n* = 12), and incubated in different media (Tyrode’s, G67-1P, and G67-10P). After 3 hours of incubation at 38°C, aliquots were collected for proteomics, metabolomics, computer-assisted sperm analysis (CASA), and flow cytometry analyses.

### CASA

Sperm motility and kinematics were assessed using CASA, (ISAS Proiser, Spain) following standard protocols (Ortega-Ferrusola *et al.* 2009). 5 μL stallion spermatozoa diluted to 25–30 × 10^6^ spermatozoa/mL were loaded in a 20 μm depth chamber (Leja®, The Netherlands) and placed on a stage warmed to 37°C. Analysis was performed on 60 consecutive digitized images, with a frame rate 60 Hz, obtained using a 10× negative phase-contrast objective (Olympus CX 41). At least 500 spermatozoa per sample were analyzed in random fields. Spermatozoa with an average velocity (VAP) >35 μm/s were considered motile. Spermatozoa deviating <45% from a straight line were classified as linearly motile. Other parameters studied included curvilinear velocity (VCL μm/s), defined as the timeline average velocity of a sperm head along its actual trajectory, the straight-line velocity (VSL μm/s), the velocity calculated along a straight line between the first and last points of the path and velocity along the average path (VAP μm/s) as the time-averaged velocity calculated along the average path.

### Flow cytometry

Flow cytometry analyses were conducted in a CytoFLEX^®^ LX flow cytometer equipped with ultraviolet (355 nm), violet (405 nm), blue (488 nm), yellow-green (561 nm), red (638 nm), and infrared (808 nm) lasers. The instrument was calibrated daily using specific calibration beads provided by the manufacturer. A compensation overlap was performed before each experiment. Files were exported as FCS files and analyzed in Cytobank© software (Beckman Coulter, USA). Unstained, single-stained, and fluorescence minus one (FMO) controls were used to determine compensations and positive and negative events, and to set regions of interest as described in previous publications by our laboratory (Gallardo Bolanos *et al.* 2014, Martin Munoz *et al.* 2015).

### Five color assessment of sperm function

A five-color panel was created to simultaneously assess a wide range of sperm components and functions, including viability, membrane permeability, mitochondrial membrane potential, mitochondrial mass, and apoptotic changes (increased membrane permeability (Gallardo Bolanos *et al.* 2014)). Stallion spermatozoa were stained with Annexin-V Alexa fluor 350 conjugate (Thermo Fisher; Ex/Em (nm) 2 μM; 361/487, Ex/Em(nm)), YoPro1 (Thermo Fisher Y3603) (12.5 nM, Ex/Em(nm) 491/509), tetrametylrhodamine TMRM (Thermo Fisher I34361) (50 nM; Ex/Em (nm) 548/574); MitoTracker Deep Red (Thermo Fisher M46753) (50 nM; Ex/Em (nm) 644/665) and ViaKrome 808 (Beckman Coulter C36628 2 μL/sample; Ex/Em(nm) 854/878). Samples were incubated in the dark at 37°C for 20 min, then washed, resuspended in PBS, and run in the flow cytometer.

### Protein solubilization

Isolated spermatozoa (200 × 10^6^ spermatozoa) were solubilized in lysis buffer 25 mM C7:C7Bz0 (3-(4-heptyl) phenyl-(3hydroxypropyl) dimethylammoniopropanesulfonate), 7 M urea, 2 M thiourea and 40 mM Tris (pH 10.4); 20 microliters of lysis buffer was added per 10 × 10^6^ spermatozoa vortexed and incubated under constant rotation at −4°C for 1 h.

### Protein quantification

Protein quantification was performed using the 2-D Quant Kit (GE Healthcare, Spain) following the instructions of the manufacturer.

### In-solution trypsin digestion

100 μg protein was taken from every sample according to its protein concentration. Type I water was used to balance all samples at a final volume of 300 μL. Then, 25 μL 100 mM ammonium bicarbonate buffer pH 8.5 was added to each sample. In this solution, the proteins were reduced by adding 1.5 μL 200 mM DTT and incubated at 56°C for 20 min. Then, the proteins were alkylated by adding 3 μL 200 mM IAA and incubating for 45 min at room temperature in the dark. Before trypsinization, samples were incubated for 20 min with 1 μL 200 mM DTT to neutralize any excess IAA. Finally, digestion was performed by adding 2 μL Trypsin Proteomics Grade (Sigma) (Trypsin solution: 1 mg/mL in 1 mM HCl) and incubating overnight at 37°C. The reaction was stopped with 1 μL 0.1% and samples were centrifuged for 5 min at 7,000 ***g***.

Finally, supernatants were dried using a centrifugal vacuum concentrator (Gyrozen, South Korea; 537 ***g***/37°C/1 mbar/3 h). The dry samples were resuspended in 20 μL mass spectrometry mobile phase, consisting of water/acetonitrile/formic acid (94.9:5:0.1).

### UHPLC-MS/MS analysis

The separation and analysis of the samples were performed with a UHPLC/MS system consisting of an Agilent 1290 Infinity II Series UHPLC (Agilent Technologies, USA) equipped with an automated multisampler module and a High-Speed Binary Pump, and coupled to an Agilent 6550 Q-TOF Mass Spectrometer (Agilent Technologies, USA) using an Agilent Jet Stream Dual electrospray (AJS-Dual ESI) interface. The control of the HPLC and Q-TOF was made by the MassHunter Workstation Data Acquisition software (Agilent Technologies, Rev. B.06.01). The sample was injected onto an Agilent AdvanceBio Peptide Mapping HPLC column (2.7 mm, 150′ 2.1 mm, Agilent Technologies), thermostated at 55°C at a flow rate of 0.4 mL/min. This column is suitable for peptide separation and analysis. The gradient program started with 2% of B (buffer B: water/acetonitrile/formic acid, 10:89.9:0.1) that stayed 5 min in isocratic mode and then it increased linearly up to 45% B in 40 min, and then, it increased up to 95% B in 15 min and it remained constant for 5 min. After this 70 min run, 5 min of post-time were followed by using the initial condition for the conditioning of the column for the next run. The mass spectrometer was operated in the positive mode. The nebulizer gas pressure was set to 35 psi, the drying gas flow was set to 10 L/min at 250°C, and the sheath gas flow was set to 12 L/min at 300°C. The capillary spray, fragmentor and octopole RF Vpp voltages were 3,500, 340, and 750 V, respectively. Profile data were acquired for both MS and MS/MS scans in extended dynamic range mode. MS and MS/MS mass range were 50–1,700 *m*/*z* and scan rates were 8 spectra/sec for MS and 3 spectra/sec for MS/MS. Auto MS/MS mode was used with precursor selection by abundance and a maximum of 20 precursors selected per cycle. A ramped collision energy was used with a slope of 3.6 and an offset of −4.8. The same ion was rejected after two consecutive scans.

### Data processing

Data processing and analysis was performed using a Spectrum Mill MS Proteomics Workbench (Rev B.04.01, Agilent Technologies, USA). Briefly, raw data were extracted under default conditions as follows: non-fixed or variable modifications were selected; (MH)+ 50–10,000 *m*/*z*; maximum precursor charge +5; retention time and *m*/*z* tolerance ±60 s; minimum signal-to-noise MS (S/N) 25; and finding ^12^C signals. The MS/MS search against the appropriate and updated protein database (in this case, Uniprot/Horse) was performed with the following criteria: non-fixed modifications were selected and as variable modification, carbamidomethylated cysteines were selected; tryptic digestion with five maximum missed cleavages; ESI-Q-TOF instrument; minimum matched peak intensity 50%; maximum ambiguous precursor charge +5; monoisotopic masses; peptide precursor mass tolerance 20 ppm; product ion mass tolerance 50 ppm; and calculation of reversed database scores.

### Validation of sperm proteins

The autovalidation strategy used was auto-threshold in the Spectrum Mill MS Proteomics Workbench (Rev B.04.01, Agilent Technologies, USA), in which the peptide score was automatically optimized for a target % false discovery rate (FDR) (1.2%). Then, the protein polishing validation was performed to increase the sequence coverage of validated results with the restriction of a new maximum target protein FDR (0%).

### Metabolomics

Samples were washed twice in PBS (600 g × 10′), then the pellet formed by the spermatozoa was frozen immediately in liquid nitrogen and kept frozen at −80°C until analysis. The sperm pellet was then re-extended in 300 μL Milli-Q water/methanol and sonicated for 3 s. Immediately afterward, it was centrifuged at 6,452 ***g*** at 4°C for 3 min and the supernatant was injected into the UHPLC-MS/MS. These metabolites were analyzed by a UHPLC/MS/MS system consisting of an Agilent 1290 Infinity II Series HPLC (Agilent Technologies, USA) equipped with an automated multi-sampler module and a High-Speed Binary Pump, coupled to an Agilent 6,470 QqQ Mass Spectrometer (Agilent Technologies, USA) using an Agilent Jet Stream Dual electrospray (AJS-Dual ESI) interface. The HPLC and QqQ detector were controlled using the MassHunter Workstation Data Acquisition software (Agilent Technologies, Rev. B.08.00). The sample was injected into an Agilent HILIC-Z HPLC column (4.6 mm, 100 mm 2.1 μm, Agilent Technologies), thermostated at 35°C, at a flow rate of 0.4 mL/min. The injection volume was 7 μL. For gradient elution, solvent A was 10 mM ammonium acetate at pH 9 in Milli Q water, and solvent B was 10 mM ammonium acetate at pH 9 in Milli Q water:acetonitrile 10:90. To start with, 98% solvent B was maintained until 5 min. Solvent B was then decreased from 98 to 60% from 5 to 10 min and held at 60% for an additional 2 min. Then, solvent B returned to the initial conditions for up to 15 min. The mass spectrometer was operated in negative mode and was run in MS/MS mode. Nitrogen was used as a nebulizing gas, drying gas, sheath gas, and collision gas. The nebulizer gas pressure was set to 35 psi, whereas the drying gas flow was set to 12 L/min at a temperature of 250°C, and the sheath gas flow was set to 15 L/min at a temperature of 350°C. The capillary spray and fragmenting voltages were 3,500 and 100 V, respectively. The multiple reaction monitoring (MRM) conditions were optimized by injecting a standard solution of each metabolite at different collision energies. Data processing and analysis were performed using the MassHunter Quantitative Analysis software (Rev B.07.00.201, Agilent Technologies, USA). Data were normalized to the number of spermatozoa.

### Image flow cytometry

Indirect immunofluorescence was performed as previously described (Tapia *et al.* 2012). After blocking, cells (175.000 spz/mL) were incubated with primary antibodies anti-LDHA, LDHB, and LDHC overnight at 4°C, diluted 1/250 in PBS containing 5% BSA (w/v). The following day, cells were washed with PBS and further incubated for 1 h at room temperature (RT) with donkey anti-rabbit IgG antibody conjugated with the Alexa Fluor 488 diluted to 1/400 in PBS containing 5% BSA (w/v). Finally, cells were thoroughly washed with PBS. A total of 5,000 cells were analyzed in the ImageStream X Mark II Imaging Flow Cytometer (Merck Millipore) using a laser of 642 nm line with intensity set to 100 mW at 60× of magnification. Data analysis of the raw images was accomplished using the IDEAS1software (Version 6.0.309). The absence of nonspecific staining was determined by processing the samples without primary antibody (secondary antibody only).

### Western blotting (WB)

To separate the proteins according to their apparent molecular masses, SDS-PAGE was performed as previously described (Aparicio *et al.* 2016). In brief, proteins were extracted and denatured by boiling for 10 min at 70°C in a loading buffer supplemented with 5% mercaptoethanol. The protein content was calculated using the Bradford assay (Bradford 1976). 30 micrograms of sperm protein extract were loaded and resolved by SDS-PAGE on a 10% polyacrylamide gel. Immunoblotting was performed by incubating the membranes in blocking buffer (milk/TBST) at 4°C overnight with anti-LDHA (1:1,500), anti-LDHB (1:1,000), and anti-LDHC (1:7,000) primary antibodies. Anti-tubulin was used as loading control 1: 42.0000 2 h at RT in 5% skim dry milk/TBST. The secondary antibody used was anti-mouse IgM 1.3,000, 30 min in 5% skim dry milk/TBST.

### Statistical analysis

All experiments were repeated at least three times with independent biological replicates. The normality of the data was assessed using the Kolmogorov–Smirnoff test. One-way ANOVA followed by Tukey’s multiple comparisons test were performed using the GraphPad Prism version 10.3.0 (www.graphpad.com) for Mac.

## Results

### Sperm motility and velocities

**Figure 1:**
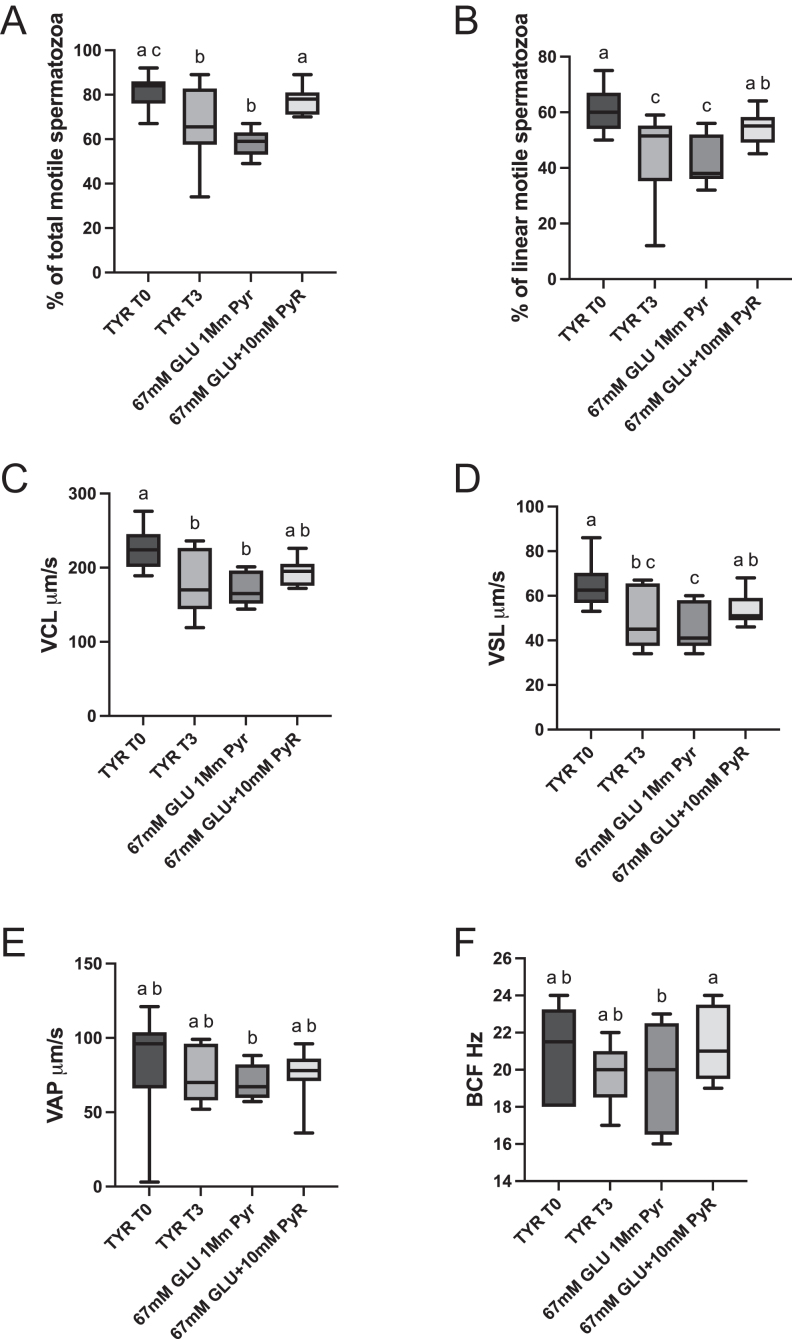
Effect of media composition on sperm motility and kinematics. Individual ejaculates were processed as described in material and methods and split samples were incubated for up to 3 h in different media. (A) Percentage of total motile spermatozoa, (B) percentage of linear motile spermatozoa, (C) circular velocity in μm/s, (D) straight line velocity (μm/s), (E) average velocity (μm/s), (G) BCF (Hz), TYR T0 = Tyrode’s beginning of the incubation period, TYR T3 = Tyrode’s after 3 h of incubation, 67 mM glucose 1 mM pyruvate after 3 h of incubation = 67 mM glucose + 1 mM pyruvate, 67 mM glucose + 10 mM pyruvate, 67 mM glucose and 10 mM pyruvate after 3 h of incubation, a-b-c- columns with different superscripts differ significantly *P* < 0.01.

Split ejaculates were incubated at 38°C for up to 3 h in different media, Tyrode’s basal media, and two variants, one containing 67 mM glucose 1 mM pyruvate and the other containing 67 mM glucose and 10 mM pyruvate. Aliquots extended in the high glucose media (67 mM glucose) after 3 h of incubation at 37°C experienced a significant drop in motility respect initial values (58.1 ± 1.8% vs 81.2 ± 1.8%; *P* < 0.0001; Fig. 1A), while aliquots incubated in the 67 mM glucose 10 mM pyruvate media maintained the motility all along the incubation period (77.1 ± 1.4%; Fig. 1A). The percentage of linear motile spermatozoa followed the same trend as total motility, with the aliquots extended in the 67 mM glucose 10 mM pyruvate media maintaining the initial linear motility (Fig. 1B). The sperm velocities were higher in the 67 mM glucose 10 mM pyruvate media, with the worst results observed in the 67 mM glucose 1 mM pyruvate media (Fig. 1C, D, E). The curvilinear velocity (VCL) was 225.5 ± 12.4 μm/s, dropping after 3 h in aliquots incubated in the 67 mM glucose media to 171.4 ± 7.5 μm/s (Fig. 1C; *P* < 0.01), while the aliquots stored in the 67 mM glucose 10 mM pyruvate media conserved the initial motility (193 ± 5.9 μm/s, Fig. 1C). The straight line (VSL) and average velocities (VAP) followed the same tendency as VCL (Fig. 1D and E). After 3 h of incubation, the beat cross frequency (BCF) was higher in the spermatozoa incubated in the 67 mM glucose 10 mM pyruvate than in the 67 mM glucose (Fig. 1F; *P* < 0.05).

### Sperm viability

#### Intact functional membranes

**Figure 2:**
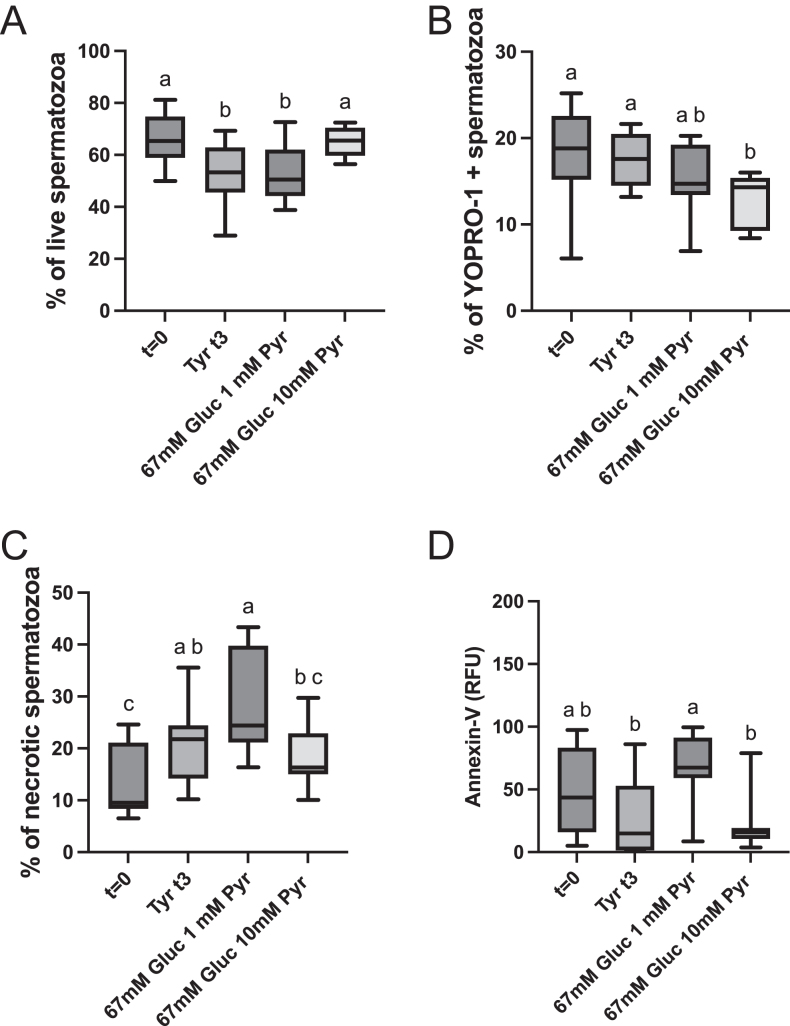
Effect of media composition on sperm viability, membrane permeability, and apoptotic changes. Individual ejaculates were processed as described in material and methods, split samples were incubated for up to 3 h in different media, and flow cytometry analysis was conducted. (A) Percentage of live spermatozoa, (B) percentage of spermatozoa with increased membrane permeability, (C) percentage of necrotic spermatozoa, (D) expression of phosphatidylserine (PS) in the outer leaflet of the sperm membranes (relative fluorescence units), TYR T0 = Tyrode’s beginning of the incubation period, TYR T3 = Tyrode’s after 3 h of incubation, 67 mM glucose 1 mM pyruvate after 3 h of incubation = 67 mM glucose + 1 mM pyruvate, 67 mM glucose + 10 mM pyruvate, 67 mM glucose and 10 mM pyruvate after 3 h of incubation, a-b-c- columns with different superscripts differ significantly *P* < 0.01.

The percentage of live spermatozoa dropped along the incubation period in aliquots extended in the Tyrode’s basal media and the 67 mM glucose 1 mM pyruvate media. At the beginning of the incubation period, the percentage of live spermatozoa was 67.6 ± 2.0%, while in the aliquots extended in the Tyrode’s basal medium and the 67 mM glucose medium were 47.5 ± 3.7% and 48.4 ± 3.5%, respectively (Fig. 2A *P* < 0.01 and *P* < 0.001). However, spermatozoa extended in the 67 mM glucose 10 mM pyruvate medium maintained the initial viability (Fig. 2A) all along the incubation period.

### Increased permeability of the membranes and spermatozoa showing phosphatidylserine (PE) transposition to the outer leaflet of the membrane

The percentage of YoPro1+ spermatozoa was reduced in aliquots extended in the 67 mM glucose 10 mM pyruvate (Fig. 2B; *P* < 0.05). In addition, this group had a reduced percentage of spermatozoa, showing phosphatidylserine transposition to the outer leaflet of the plasma membrane (Fig. 2D; *P* < 0.001).

### Necrotic spermatozoa

The spermatozoa extended in the 67 mM glucose 1 mM pyruvate media presented the highest percentage of necrotic spermatozoa (Viakrome 808 positive, 28.3 ± 2.2%) after 3 h of incubation, while the addition of 10 mM pyruvate to the 67 mM glucose media reduced the percentage of necrotic spermatozoa to 18.1 ± 1.3% (Fig. 2C; *P* < 0.001).

### Mitochondrial membrane potential and mitochondrial mass

**Figure 3:**
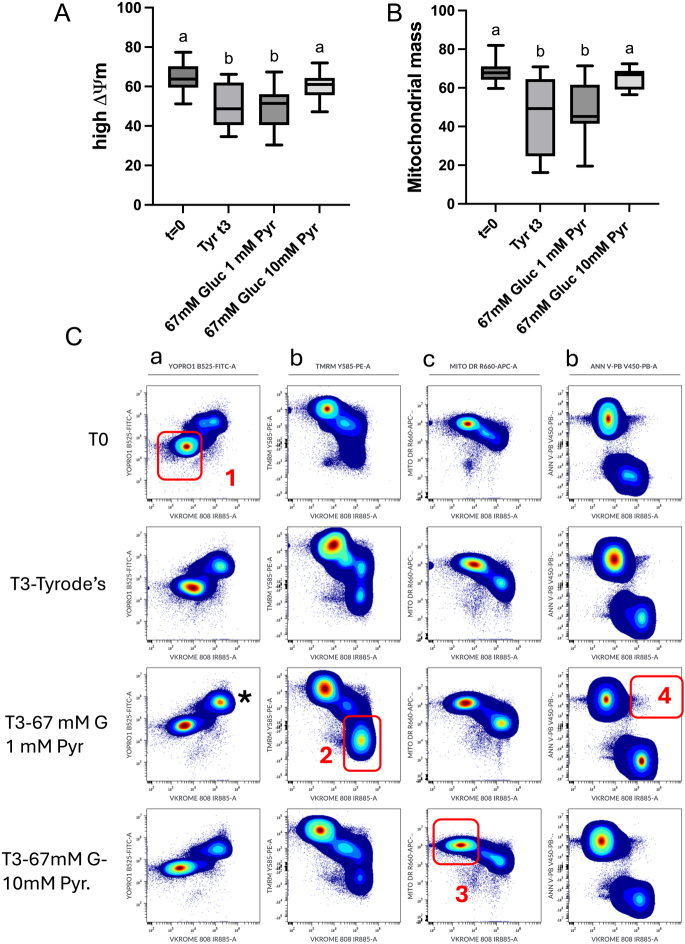
Effect of media composition on the percentage of spermatozoa with high mitochondrial membrane potential and mitochondrial mass. Individual ejaculates were processed as described in material and methods. Split samples were incubated for up to 3 h in different media, and flow cytometry analysis was conducted. (A) Percentage of spermatozoa showing high mitochondrial membrane potential, (B) changes in mitochondrial mass along the incubation period in different media, (C) flow cytometry dot plots showing the five-color panel in concatenated replicates. Density plots are presented. In column a, population 1 is the percentage of live spermatozoa, note that the density plot indicates an increase in the population of dead spermatozoa in spermatozoa incubated in the 67 mM glucose 1 mM pyruvate media (*). Column B dot plots showing changes in the mitochondrial membrane potential; gate 2 represents the population of dead spermatozoa with low mitochondrial membrane potential. Column C dot plots showing changes in the mitochondrial mass; population 3 shows live spermatozoa maintaining mitochondrial mass. Column C dot plots showing the transposition of phosphatidylserine (PS) to the outer membrane, a population of live spermatozoa with PS translocation to the outer membrane is depicted in 4. TYR T0 = Tyrode’s beginning of the incubation period, TYR T3 = Tyrode’s after 3 h of incubation, 67 mM glucose 1 mM pyruvate after 3 h of incubation = 67 mM glucose + 1 mM pyruvate, 67 mM glucose + 10 mM pyruvate, 67 mM glucose and 10 mM pyruvate after 3 h of incubation, a-b-c- columns with different superscripts differ significantly *P* < 0.01.

Both mitochondrial mass and mitochondrial membrane potential decreased over the incubation period (Fig. 3). However, this reduction was not present in the spermatozoa incubated in the 67 mM glucose 10 mM pyruvate media after 3 h of incubation (64.5 ± 1.5% vs 60.3 ± 1.6% n.s), in which both parameters were similar to the initial values and significantly higher than spermatozoa incubated in the other two media (50.5 ± 2.45%) *P* < 0.01 vs Tyrode’s and *P* < 0.001 (49.1 ± 2.2%) vs 67 mM glucose 1 mM pyruvate; Fig. 3A). Concerning the mitochondrial mass, the tendency observed was similar, with a reduction of the mitochondrial mass after 3 h of incubation in spermatozoa extended in basal Tyrode’s and 67 mM glucose 1 mM pyruvate (*P* < 0.0001), while the mitochondrial mass was maintained after 3 hours of incubation in spermatozoa in the 67 mM glucose 10 mM pyruvate media (Fig. 3B).

### Analysis of the metabolic proteome

**Figure 4:**
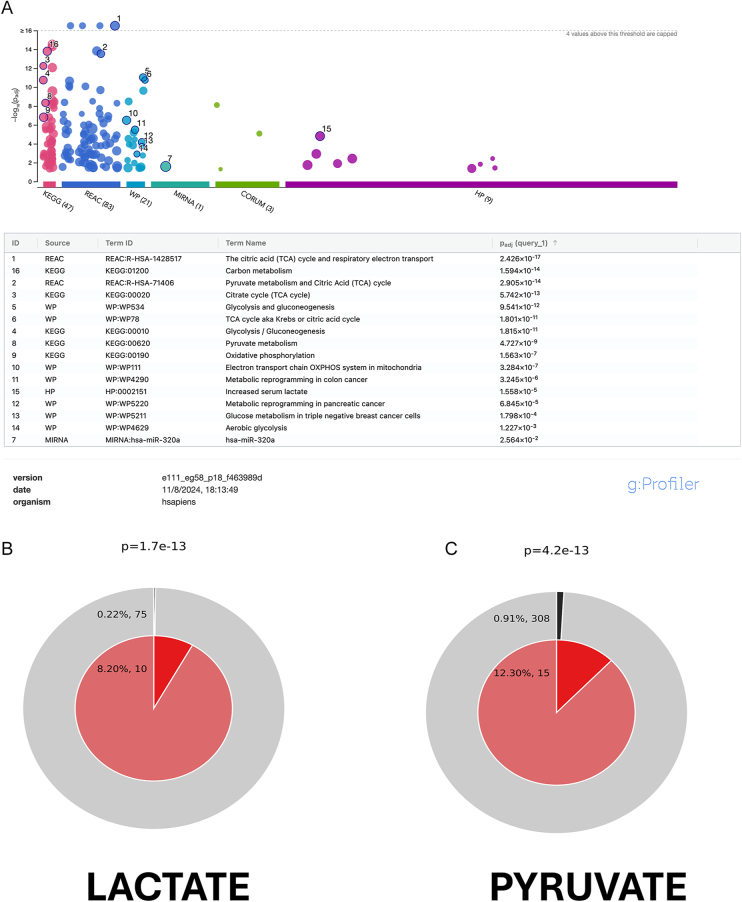
Enrichment analysis of the metabolic proteins detected in stallion spermatozoa. Equine orthologs were transformed into human orthologs and analyzed in the g profiler (https://biit.cs.ut.ee/gprofiler/gost) using default settings and in metascape (https://metascape.org/gp/index.html#/main/step1) using custom analysis interrogating the list of proteins against the terms ‘pyruvate’ and ‘lactate’. (A) Manhattan plot showing enriched terms, (B and C) enrichment of proteins matching membership terms: LACTATE and PYRUVATE. The outer pie shows the number and percentage of genes in the background associated with the membership (in black); the inner pie shows the number and percentage of genes in the individual input gene list associated with the membership. The *P*-value indicates whether the membership is statistically significantly enriched in the list.

**Figure 5:**
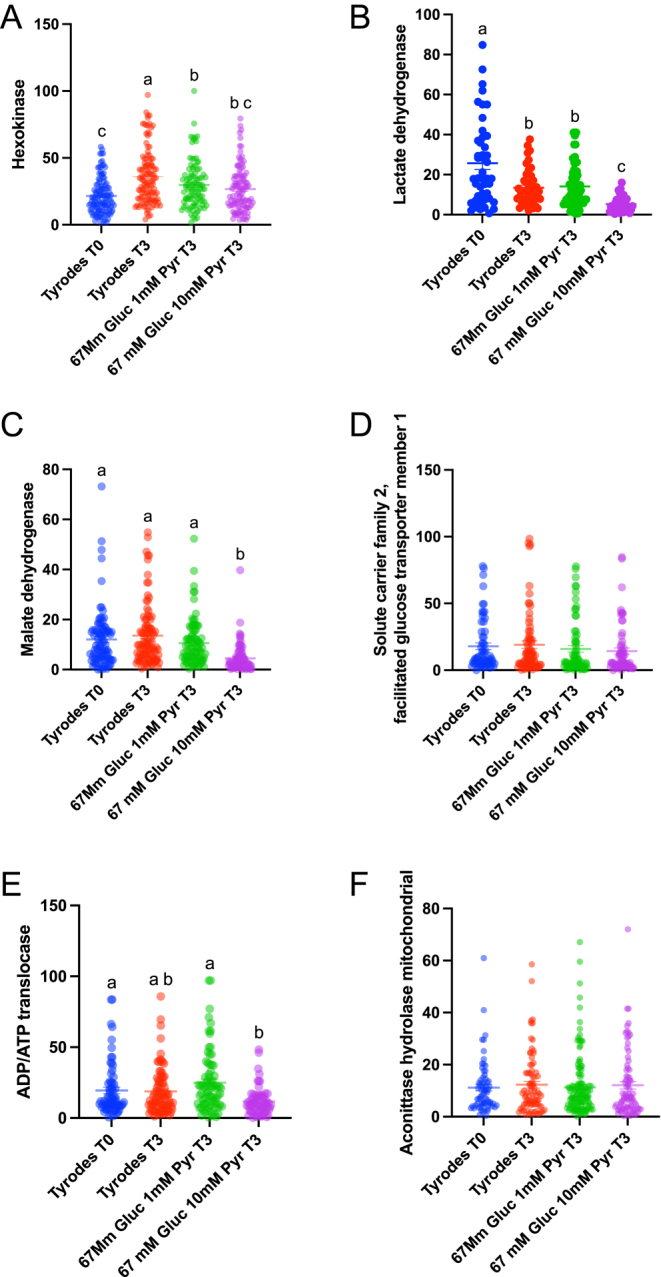
Changes in the relative amounts of metabolic proteins in spermatozoa incubated in different media. Individual ejaculates were processed as described in material and methods. Split samples were incubated for up to 3 h in media containing different amounts of glucose and pyruvate, and proteomic analysis was performed as described in material and methods. TYR T0 = Tyrode’s at the beginning of the incubation period (T0), TYR T3 = Tyrode’s after 3 h of incubation, 67 mM glucose 1 mM pyruvate after 3 h of incubation = 67 mM glucose + 1 mM pyruvate, 67 mM glucose and 10 mM pyruvate after 3 h of incubation = 67 mM glucose + 10 mM pyruvate. Values with different superscripts differ statistically a-c *P* < 0.001.

**Figure 6:**
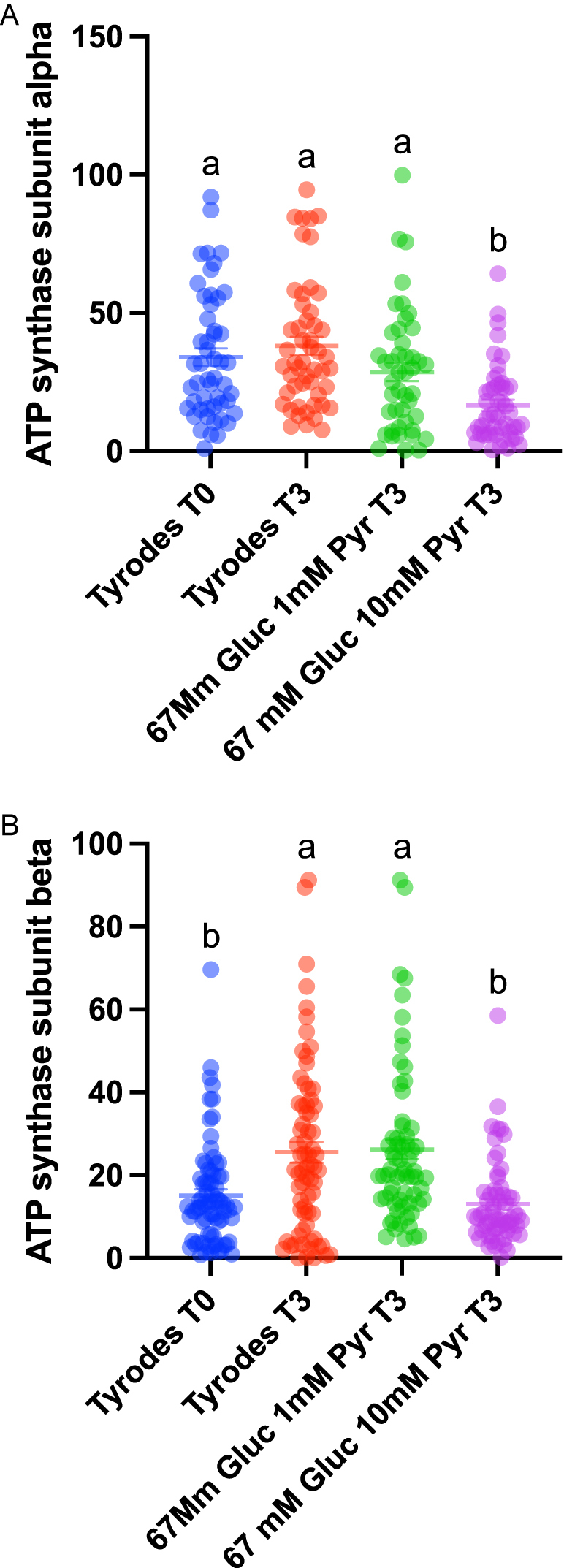
Changes in the relative amounts of ATP synthase subunits alpha (A) and beta (B) along the incubation period in stallion spermatozoa. Individual ejaculates were processed as described in material and methods. Split samples were incubated for up to 3 h in different media, and proteomic analysis was performed as described in material and methods. TYR T0 = Tyrode’s at the beginning of the incubation period (T0), TYR T3 = Tyrode’s after 3 h of incubation, 67 mM glucose 1 mM pyruvate after 3 h of incubation = 67 mM glucose + 1 mM pyruvate, 67 mM glucose and 10 mM pyruvate after 3 h of incubation = 67 mM glucose + 10 mM pyruvate. Values with different superscripts differ statistically a-c *P* < 0.001.

**Figure 7:**
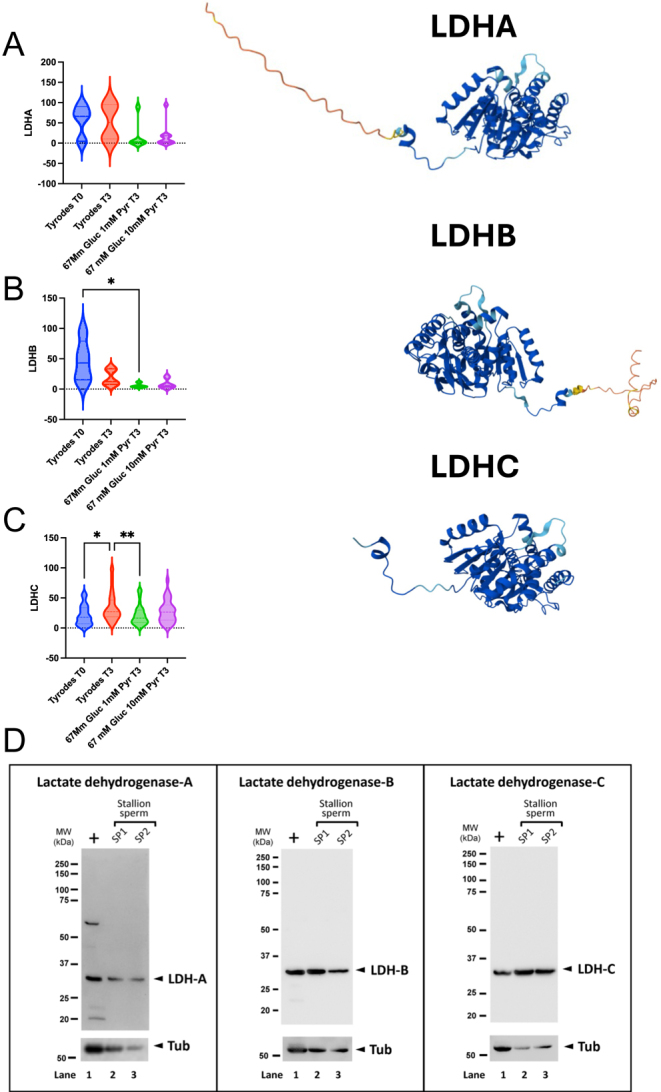
Changes in the relative amounts of the three isoforms of LDH detected in stallion spermatozoa incubated up to 3 h in media with different concentrations of glucose and pyruvate. TYR T0 = Tyrode’s at the beginning of the incubation period (T0), TYR T3 = Tyrode’s after 3 h of incubation, 67 mM glucose 1 mM pyruvate after 3 h of incubation = 67 mM glucose + 1 mM pyruvate, 67 mM glucose and 10 mM pyruvate after 3 h of incubation = 67 mM glucose + 10 mM pyruvate. The structure of the proteins was downloaded from AlphaFold https://www.alphafold.ebi.ac.uk. (A) LDHA, (B) LDHB, (C) LDHC *P < 0.05, **P < 0.01, (D) Identification of the three isoforms of LDH A, B, and C in stallion sperm lysates.

**Figure 8:**
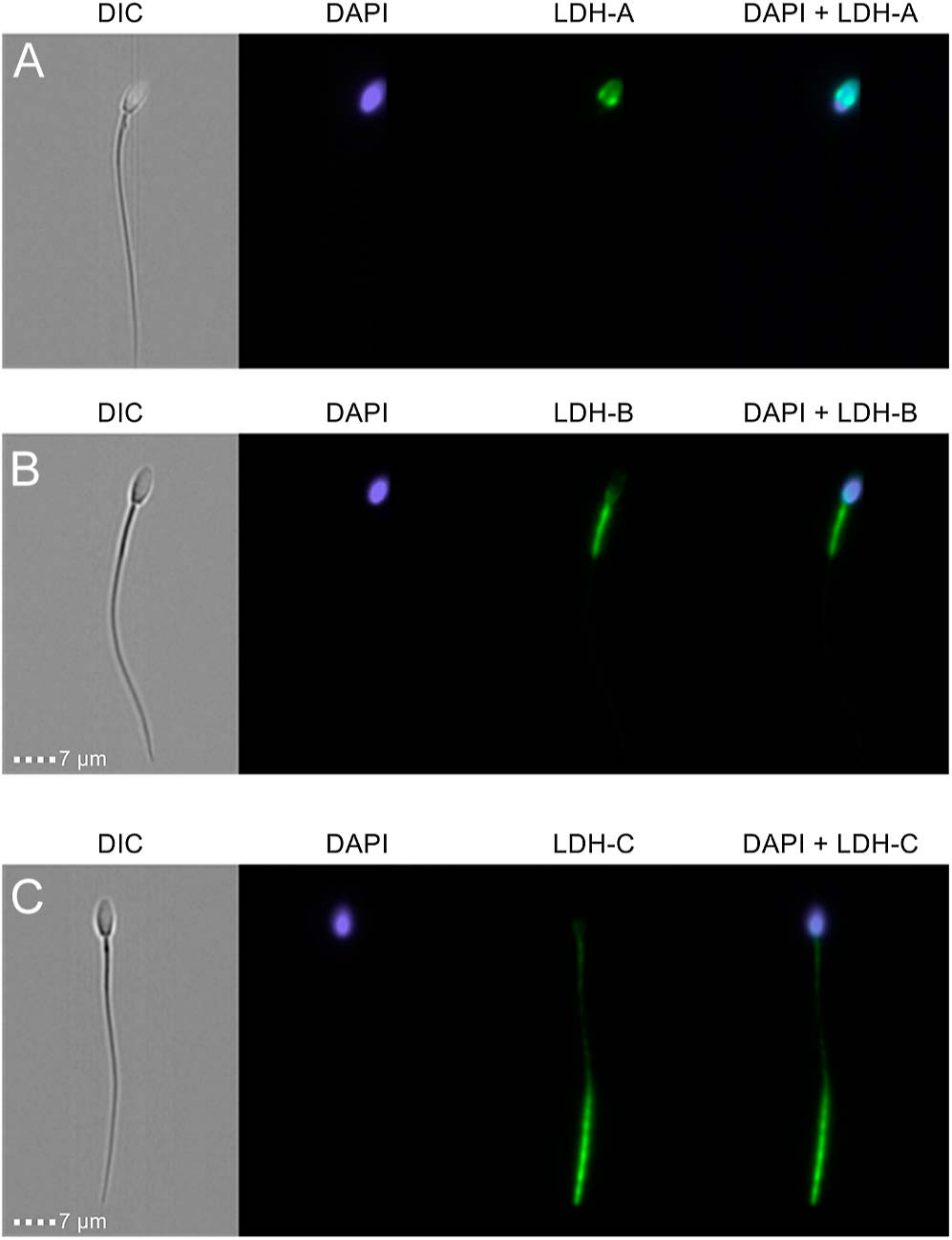
Immunolocalization of the three isoforms of LDH in stallion spermatozoa. Stallion spermatozoa were processed as indicated in material and methods and evaluated using image flow cytometry (A) LDHA, this protein is expressed in the acrosomal region (B) LDH B is expressed in the midpiece, over the mitochondrial sheath (C) LDHC is expressed in the tail, especially in the principal piece.

Using strict criteria to reach a 0% FDR in the protein search, we identified 164 proteins (Supplementary Table 2). Equine orthologs were transformed into human orthologs in the g profiler (https://biit.cs.ut.ee/gprofiler/gost), and enrichment analysis was performed. Interestingly, the reactome pathways REAC:R-HAS-71406 (the citric acid cycle and respiratory electron transport; *P* = 2.426 × 10^−17^), REAC:R-HAS-71406 (Pyruvate metabolism and TCA cycle; *P* = 2.905 × 10^−14^) and the WP pathway WP4629 (aerobic glycolysis; *P* = 1.227 × 10^−3^) were highly enriched in stallion spermatozoa (Fig. 4A). We performed a custom analysis in metascape (Zhou *et al.* 2019), using the terms ‘pyruvate’ and ‘lactate’ to study its enrichment. Both terms were highly enriched in the stallion spermatozoa; LACTATE *P* = 1.7 × 1e^−13^ and PYRUVATE *P* = 4.2 × e^−13^ (Fig. 4B and C). Then, we analyzed differences among groups in the major metabolism-related proteins identified in all the samples. The proteins studied were hexokinase, lactate dehydrogenase, malate dehydrogenase, solute carrier family 2, facilitated transporter member 1, ADP/ATP translocase, aconitase hydrolase mitochondrial and ATP synthase units α and β. We observed differences in the amounts of hexokinase (Fig. 5A) that were detected in higher amounts after 3 h of incubation in samples extended in the Tyrode`s and in the 67 mM glucose media (*P* < 0.001 and *P* < 0.01, respectively). Significant differences were also observed in the amounts of lactate dehydrogenase (Fig. 5B), with the lowest quantity of this protein detected in the 67 mM glucose 10 mM pyruvate group (*P* < 0.001). In this group, we also observed reduced amounts of malate dehydrogenase (Fig. 5C, *P* < 0.01), ADP/ATP translocase (*P* < 0.01; Fig. 6E), and ATP synthase subunits α and β (*P* < 0.01; Fig. 7A and B). Concerning LDH, three isoforms were detected (Figs 7 and 8): LDHA (cytosolic), LDHB (mitochondrial, with higher affinity for lactate), and LDHC (cytosol, motile cilium). When the relative amounts of the different isoforms were evaluated, reductions were observed in the 67 mM glucose 1 mM pyruvate group in LDHB and C (Fig. 7B and C *P* < 0.05).

### Subcellular location of LDH isoforms

The location of the three LDH isoforms identified in the proteomic study and confirmed using western blotting was investigated using image flow cytometry (Fig. 8). LDHA was identified in the sperm head (Fig. 8A), in the acrosome, LDHB was identified in post-acrosomal region and principally in the mitochondrial region of the midpiece (Fig. 8B) and LDHC was identified all along the sperm tail, midpiece, and especially in the principal piece and the distal region of the flagella (Fig. 8C).

### Inhibition studies

**Figure 9:**
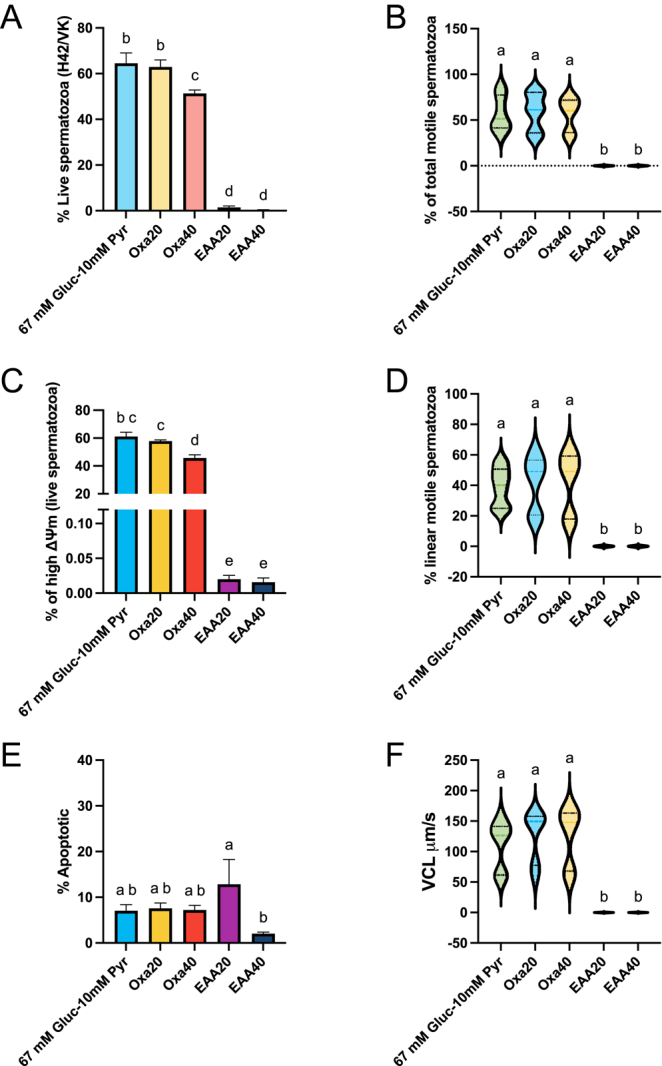
Inhibition assay using oxamate (LDHA inhibitor) and EAA, a specific inhibitor of the C isoform of LDHC. Three independent ejaculates from three different stallions were used in the experiment (*n* = 9). Stallion spermatozoa were incubated for up to 3 h in the presence of 0, 20, and 40 mM oxamate and 0, 20, and 40 mM EAA. After 3 h of incubation, motility and circular velocity (VCL μm/s) were measured using CASA. The percentages of viable, apoptotic, and high mitochondrial potential spermatozoa were investigated using flow cytometry, b-d *P* < 0.01; b-c-d-e *P* < 0.00001, a-b *P* < 0.01.

We used two different inhibitors of LDH, oxamate and ethylaminooxoacetic acid (EAA), 0, 20 and 40 mM. While oxamate inhibits preferentially LDHA (Altinoz & Ozpinar 2022), EAA is a specific inhibitor of the LDHC isoform (He *et al.* 2016, Tan *et al.* 2022). Aliquots of stallion spermatozoa extended in a 67 mM glucose −10 mM pyruvate supplemented, with 0, 20, and 40 mM of the two inhibitors incubated for up to 3 h at 38°C. At the beginning and the end of the incubation period, aliquots were taken for sperm analysis, CASA, and flow cytometry. Oxamate at 40 mM reduced the percentage of live spermatozoa and spermatozoa with high mitochondrial membrane potential after 3 h of incubation at 37°C without affecting the sperm motility and curvilinear velocity. However, the specific inhibitor of LDHC, EAA, inhibited sperm motility, depolarized mitochondria, and caused sperm death (Fig. 9A, B, C, D, E, F; *P* < 0.0001).

### Metabolic analysis

**Figure 10:**
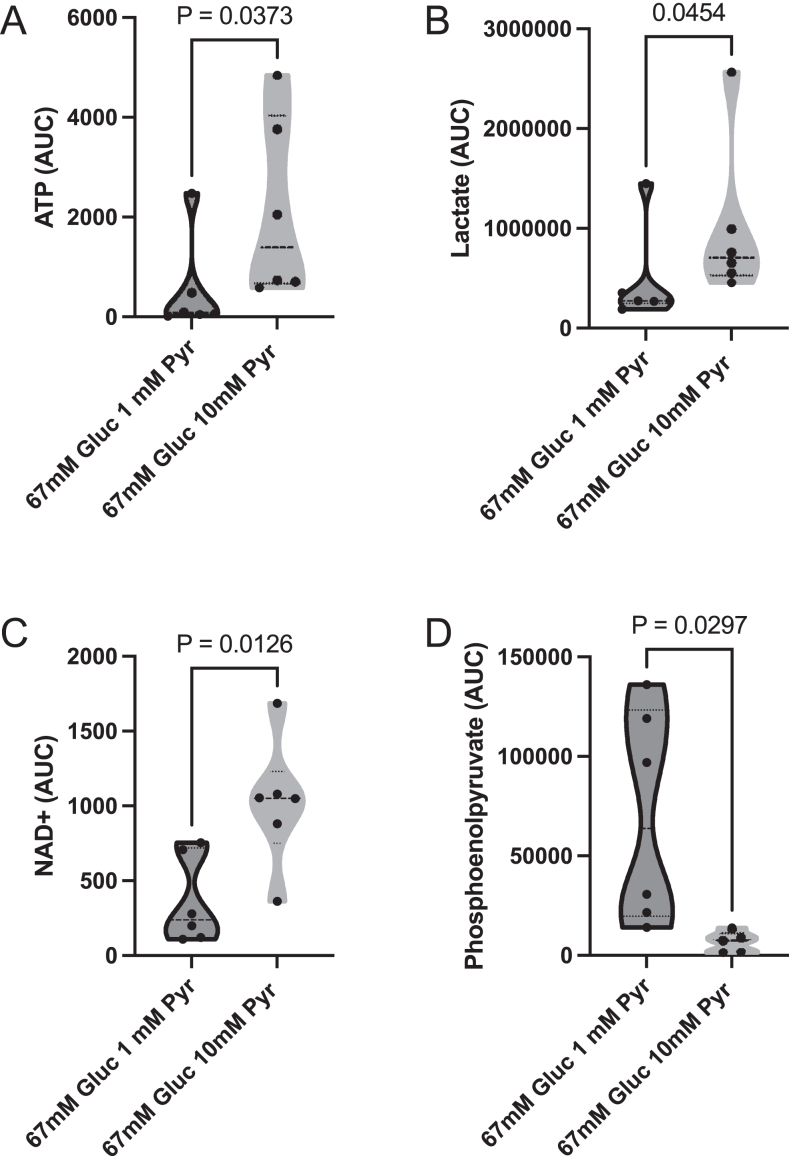
UHPLC/MS/MS analysis of changes in the relative amounts of lactate, NAD+, ATP, and phosphoenolpyruvate in stallion spermatozoa incubated in two media differing in the amount of pyruvate, 67 mM glucose and 1 mM pyruvate, and 67 mM glucose and 10 mM pyruvate.

We performed mass spectrometry-based metabolic analysis to disclose the metabolic mechanism, improving sperm quality in the 67 mM glucose 10 mM pyruvate group. We focused on relative changes in lactate and NAD^+^ as indicators of LDH activity, phosphoenolpyruvate as an indicator of glycolysis efficiency, and ATP as an indicator of the global efficiency of metabolism. Adding 10 mM pyruvate instead of 1 mM pyruvate to the 67 mM glucose media increased the relative amounts of lactate, NAD^+^, and ATP and reduced phoshoenolpyruvate (Fig. 10; *P* < 0.05).

## Discussion

Supplementation with 10 mM pyruvate to a 67 mM glucose media rescued the decline in all sperm parameters observed in a 67 mM glucose medium containing only 1 mM pyruvate after 3 h of incubation at 37°C. In the group supplemented with 10 mM pyruvate, sperm parameters remained stable throughout the incubation period. In contrast, the group with 67 mM glucose and 1 mM pyruvate showed significant reduction in these parameters. Furthermore, differences in the relative amounts of proteins involved in glycolysis, the TCA cycle, and oxidative phosphorylation were observed between the groups. Notably, and for the first time, three isoforms of LDH – LDHA, LDHB, and LDHC – were identified in stallion spermatozoa. These isoforms exhibited high compartmentalization, a finding that suggests the presence of a functional intracellular lactate shuttle within stallion spermatozoa (Storey & Kayne 1977).

The positive effects of pyruvate have been previously described (O'Flaherty *et al.* 2005, Medrano *et al.* 2006, Ramirez-Agamez *et al.* 2024). While the observed effects were largely attributed to the metabolic actions of pyruvate, it also exhibits significant antioxidant properties through several interconnected mechanisms. Pyruvate directly neutralizes reactive oxygen species (ROS), such as hydrogen peroxide, via chemical reactions, acting as an electron donor due to its alpha-keto acid structure. Furthermore, it influences cellular redox balance, particularly within mitochondria, by affecting NADH/NAD^+^ ratios and preventing mitochondrial membrane potential collapse, thereby modulating ROS production. This multifaceted approach, encompassing both direct scavenging and redox modulation, allows pyruvate to effectively protect cells and mitochondria from oxidative damage (Wang *et al.* 2007, Huang *et al.* 2013, Gray *et al.* 2014, Toshniwal *et al.* 2024). This aspect may be of special relevance in the stallion spermatozoa.

Although the important role of pyruvate was acknowledged several decades ago (Hutson *et al.* 1977, Van Dop *et al.* 1977), the interest in pyruvate metabolism has renewed in recent years. Several studies reveal the importance of pyruvate on the stallion spermatozoa (Bruemmert *et al.* 2002, Gibb *et al.* 2015, Darr *et al.* 2016, Becerro-Rey *et al.* 2024, Martin-Cano *et al.* 2024). Moreover, proteomic studies underline the importance of pyruvate in the metabolism of stallion spermatozoa (Swegen *et al.* 2015, Martin-Cano *et al.* 2020, Gaitskell-Phillips *et al.* 2021, Peña *et al.* 2024*b*). Pyruvate metabolism appears highly prominent in stallion spermatozoa when considering the broader equine proteome. Our study supports this observation, showing an overrepresentation of metabolic proteins in these cells, especifically those related to ‘pyruvate’, ‘lactate’, and the particularly interesting term ‘aerobic glycolysis’.

A noteworthy finding of our study was identifying, to the author’s knowledge for the first time, the three isoforms of LDH: LDHA, LDHB, and LDHC in stallion spermatozoa. LDHA is cytosolic, LDHB is located in the intermembrane space in the mitochondria (Storey & Kayne 1978), and LDHC is located in the cytosol and the fibrous sheath of the sperm tail. In addition, these three isoforms show different affinities for the substrate. While isoforms A and C have more affinity for pyruvate, the mitochondrial isoform B shows more affinity for lactate (Dawson *et al.* 1964, Zinkham *et al.* 1964, Liang *et al.* 2016).

The finding, and the subcellular location of these three isoforms of LDH, emphasized the importance of aerobic glycolysis in the spermatozoa (Peña *et al.* 2024*a*). LDHC is present in the cytoplasm and the fibrous sheath and may represent a source of NAD^+^ to sustain glycolysis, even under reduced mitochondrial function when insufficient regeneration of NAD^+^ in the complex I of the electron transport chain (ETC) occurs. This isoform seems essential for sperm function. In our study, inhibition of this isoform caused sperm demise, while inhibition of isoform A only had a moderate effect on viability and mitochondrial membrane potential. This finding is noteworthy and consistent with previous findings in mouse models (Odet *et al.* 2011, Odet *et al.* 2013). Given the location of the LDHC isoform in the stallion spermatozoa, a reduction of motility by inhibiting this enzyme was expected.

However, our findings suggest a principal role for this isoform beyond motility, impacting sperm viability and function. Notably, previous studies investigating LDHC function in spermatozoa have predominantly used oxamate as an inhibitor (Iida-Norita *et al.* 2023). While oxamate is a mild inhibitor that preferentially inhibits the LDHA (Qiao *et al.* 2021), to the best of our knowledge, (ethylamino) (oxo)acetic acid (EAA), an inhibitor with higher affinity for LDHC (Gupta 2012, He *et al.* 2016, Tan *et al.* 2022), has not been widely employed in sperm metabolism research. We used the specific inhibitor EAA, which showed a potent inhibitory effect, further supporting the notion that LDHC plays additional roles in sperm beyond motility regulation.

Recent reports indicate a functional interaction between LDHC and LDHA in the spermatozoa (Dodo *et al.* 2019). We also found the presence of the LDHB, the mitochondrial isoform of LDH. The LDHB converts lactate into pyruvate in the intermembrane space, avoiding reductive stress in the cytoplasm due to excess NADH, while allowing the mitochondrial capture of both carbon and reducing equivalents (Chen *et al.* 2016). In this model, pyruvate can enter aerobic glycolysis, generating NAD^+^, which is essential for the glycolytic enzymes in the midpiece. Simultaneously, the lactate produced is transported into the mitochondria. There, it is oxidized back to pyruvate, which then fuels the TCA cycle, providing the reducing equivalents (NADH and FADH2) necessary for the ETC (Odet *et al.* 2011, Odet *et al.* 2013, Liu *et al.* 2019). This collaborative mechanism may explain the results of our inhibition experiment. Specifically, the EAA inhibits LDHC, leading to a depletion of NAD^+^. This NAD^+^ depletion impairs aerobic glycolysis in the flagellum, consequently compromising motility. Simultaneously, the EAA also reduces lactate levels. Since mitochondrial LDHB relies on lactate as a substrate, this reduction compromises the Krebs cycle and the availability of reducing equivalents needed for the ETC. Therefore, we propose the existence of a lactate/pyruvate shuttle in stallion spermatozoa. This shuttle functions by providing NAD^+^ in the cytoplasm to support glycolysis. The lactate produced by glycolysis is then imported into the mitochondria, where it is oxidized to pyruvate and fuels the Krebs cycle (Gallina *et al.* 1994). Lactate may be a major carbon source for the stallion spermatozoa (Darr *et al.* 2016, Becerro-Rey *et al.* 2024, Ramirez-Agamez *et al.* 2024). This mechanism may underlie the metabolic flexibility of stallion spermatozoa and their ability to adapt to varying conditions within the mare’s reproductive tract, particularly the increased energy requirements of processes such as capacitation, the acrosome reaction, and fertilization (Balbach *et al.* 2023, Mateo-Otero *et al.* 2023). In the mouse model, it has been demonstrated that transitions in motility (from activated to hyperactivated) are regulated by changes in the metabolic microenvironment. Specifically, pyruvate stimulates hyperactivation by inhibiting lactate oxidation and promoting glycolysis in the flagellum through the provision of NAD^+^(Schmidt *et al.* 2024).

The amounts detected of malate dehydrogenase, ADP/ATP translocase, and ATP synthase subunits alpha and beta were reduced in the 67 mM glucose 10 mM pyruvate group. A possible explanation is that the simultaneous increase in ATP, lactate, and NAD^+^, alongside elevated mitochondrial activity, reflects a metabolic state, where both glycolysis and mitochondrial respiration are upregulated as we propose in the lactate/pyruvate shuttle. High pyruvate concentrations may inhibit some enzymes and alter their stability, reducing their detection in mass spectrometry. The reduction of malate dehydrogenase can be linked to changes in the malate–aspartate and glycerol–phosphate electron shuttles, reflecting an adaptive shift to maximize energy production through parallel pathways (Palmieri 2013). However, the more likely explanation for these changes may be that binding to substrates or products can induce enzyme conformational changes. These changes may affect how the enzyme ionizes or how accessible its peptides are for detection, ultimately reducing its signal in mass spectrometry analysis (Konermann *et al.* 2011, Giampa & Sgobba 2020). Spermatozoa are generally considered transcriptionally silent cells, leading to the expectation that protein levels would decrease rather than increase. However, this is a subject of debate, and recent evidence suggests that spermatozoa can indeed synthesize proteins (Njoroge *et al.* 2025). Interestingly, these newly synthesized proteins were found to be involved in glutathione synthesis, which aligns with previous findings from our laboratory (Ortega-Ferrusola *et al.* 2019).

In summary, we have, for the first time, identified the presence of three LDH isoforms – LDHA, LDHB, and LDHC – in stallion spermatozoa. We propose a novel mechanism, a lactate/pyruvate shuttle, which elucidates the critical roles of lactate and pyruvate in sperm functionality. This shuttle may enhance both glycolysis and mitochondrial respiration, enabling spermatozoa to adapt their metabolism to the varying conditions encountered during their journey from ejaculation to fertilization. While further research is necessary to fully unravel the intricate metabolic network of stallion spermatozoa, our current findings highlight the remarkable plasticity of their metabolism, potentially offering avenues for improving existing reproductive biotechnologies.

## Supplementary materials



## Declaration of interest

The authors declare that there is no conflict of interest that could be perceived as prejudicing the impartiality of the work reported.

## Funding

 The authors received financial support for this study from the Ministerio de Ciencia-European Fund for Regional Development (EFRD), Madrid, Spain, grant PID2021-122351OB-I00 MCIU/AEI/10.13039/501100011033/FEDER, UE. Junta de Extremadura-FEDER (IB24001, GR24007). LBC holds a PhD grant from the Ministry of Science, Madrid, Spain (PRE2022-103090).

## Author contribution statement

L B R conducted the experiments, F E M C designed the study and conducted the experiments. C O F critically reviewed the manuscript. E da S-Á performed experiments. A S-Á performed experiments. C O-P and J A T performed experiments. M C G obtained funding and critically reviewed experiments and manuscript. F J P obtained funding, designed and administered the study, performed the formal data analysis, and drafted the manuscript. All authors read and approved the final version of the manuscript.

## Data availability

The underlying data for this article are available in the article and its online supplementary material.
