# Supplementary material for: Stallion spermatozoa express LDH isoforms A, B, and C, with LDHC playing a crucial role in sustaining sperm viability
Source: Reproduction. 2025 Jun 5;170(1):e240436. doi: 10.1530/REP-24-0436 (PMC12150302; doi:10.1530/REP-24-0436)
Supplement: Supplementary file 1 [file supplementary_materials.pdf]

Tyrode's 3% BSA<br>306 mOsm/L	
**Chemical**	**mM**
**BSA**	
**NaCl**	96
**Na Pyruvate**	1
**D-Glucose**	5
**$NaHCO_3$**	15
**Na L-Lactate**	21,7
**$CaCl_2$ * $2H_2O$**	2
**KCl**	3,1
**$MgSO_4$ * $7H_2O$**	0,4
**KH_2PO_4**	0,3
**HEPES**	20
**Penicillin G**	0,058 g/L
**Dihidrostreptomycin**	0,05 g/L

Tyrode's 3% BSA-67mM Glucose<br>306 mOsm/L	
**Chemical**	**mM**
**BSA**	
**NaCl**	**65**
**Na Pyruvate**	1
**D-Glucose**	**67**
**$NaHCO_3$**	15
**Na L-Lactate**	21,7
**$CaCl_2$ * $2H_2O$**	2
**KCl**	3,1
**$MgSO_4$ * $7H_2O$**	0,4
**KH_2PO_4**	0,3
**HEPES**	20
**Penicillin G**	0,058 g/L
**Dihidrostreptomycin**	0,05 g/L

Supplementary table 1.- Recipes of the different media used in this study

Tyrode's 3% BSA-67mM Glucose-10mM Na Pyruvate 306 mOsm/L	
**Chemical**	**mM**
**BSA**	
**NaCl**	**56**
**Na Pyruvate**	**10**
**D-Glucose**	**67**
**$NaHCO_3$**	15
**Na L-Lactate**	21,7
**$CaCl_2$ * $2H_2O$**	2
**KCl**	3,1
**$MgSO_4$ * $7H_2O$**	0,4
**KH_2PO_4**	0,3
**HEPES**	20
**Penicillin G**	0,058 g/L
**Dihidrostreptomycin**	0,05 g/L

Supplementary table 2.- List of metabolic proteins identified in our experiment
